# ICU-Acquired Weakness Complicated With Bilateral Foot Drop After Severe COVID-19: Successful Rehabilitation Approach and Long-Term Follow-Up

**DOI:** 10.7759/cureus.36566

**Published:** 2023-03-23

**Authors:** Tomoyo Taketa, Yuki Uchiyama, Norihiko Kodama, Tetsuo Koyama, Kazuhisa Domen

**Affiliations:** 1 Rehabilitation Medicine, Hyogo College of Medicine, Nishinomiya, JPN; 2 Rehabilitation Medicine, Nishinomiya Kyoritsu Neurosurgical Hospital, Nishinomiya, JPN

**Keywords:** bilateral peroneal nerve palsy, ankle-foot orthosis, polyneuropathy, electorophysiological examination, coronavirus disease 2019

## Abstract

Coronavirus disease 2019 (COVID-19) is associated with muscle and nerve injuries as a consequence of prolonged critical illness. We report here a case of intensive care unit-acquired weakness (ICU-AW) with bilateral peroneal nerve palsy after COVID-19. A 54-year-old male with COVID-19 was transferred to our hospital. He was treated by mechanical ventilation and veno-venous extracorporeal membrane oxygenation (VV-ECMO), from which he was successfully weaned. However, by day 32 of ICU admission, he had developed generalized muscle weakness with bilateral foot drop and was diagnosed with intensive care unit-acquired weakness complicated with bilateral peroneal nerve palsy. Electrophysiological examination showed a denervation pattern in the tibialis anterior muscles, indicating that the foot drop was unlikely to recover immediately. Gait training with customized ankle-foot orthoses (AFO) and muscle-strengthening exercises were started as part of a regimen that included a stay in a convalescent rehabilitation facility and outpatient rehabilitation. Seven months after onset, he returned to work, and 18 months after onset, he had improved to the same level of activities of daily living (ADLs) as before onset. Outcome prediction by electrophysiological examination, appropriate prescription of orthoses, and continuous rehabilitative treatment that focused on locomotion contributed to the successful outcome in this case.

## Introduction

Coronavirus disease 2019 (COVID-19) has become a major concern in clinical medicine since November 2019. Severe cases of COVID-19 are often treated in the intensive care unit (ICU) [1]. However, with prolonged management on a ventilator, deep sedation, steroid use, and increased risk of developing diabetes mellitus, these patients are at risk of ICU-acquired weakness (ICU-AW) [2]. In addition to ICU-AW, severe COVID-19 can be complicated with various neuromuscular complications, including stroke and peripheral neuropathy [3].

Early mobilization is important for the prevention of ICU-AW and functional improvement from neuromuscular disturbance caused by COVID-19. However, in patients with severe COVID-19, this is often hampered by poor respiratory status, the need for muscle relaxants, and limited staffing because of the need for infection control [2]. Furthermore, recovery from COVID-19 may be impeded by neuromuscular disturbance [1], which also hinders early mobilization and has a significant impact on patients’ ability to resume their normal activities of daily living (ADLs) and functional improvement [3].

This report describes a case of ICU-AW complicated with bilateral peroneal nerve palsy secondary to acute respiratory distress syndrome as a result of COVID-19. Electrophysiological examinations were performed during the subacute period. Based on outcome predictions, gait training while wearing ankle-foot orthoses (AFO) was prescribed. As a result of the early and continuous intervention, this patient returned to work and recovered the same life as before the onset of his disease [4].

## Case presentation

A 54-year-old male who was fully independent in ADL visited his doctor complaining of a fever and cough that persisted for two days. A polymerase chain reaction test of nasal and throat swabs was positive for COVID-19. He had smoked 20 cigarettes/day for approximately 25 years and was obese (height: 183 cm, weight: 105 kg, body mass index: 31.4 kg/m^2^). Comorbidities included diabetes mellitus, hypertension, and hyperlipidemia. His job as an educational administrator in the civil service required an approximately 30-minute commute by train and a walk to and from the train station on foot. In addition to his daily desk job, he was required to give a weekly one-hour lecture presentation while standing. Written consent to publish this report was obtained from the patient.

**Figure 1:**
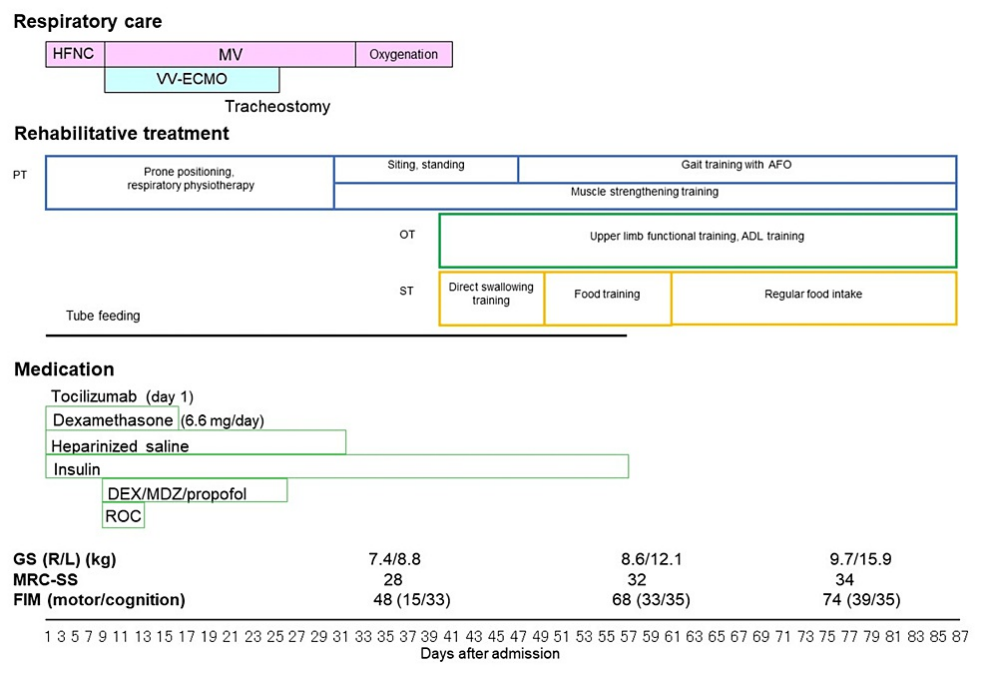
Clinical course during the acute hospitalization period Dexamethasone was continued and tocilizumab was started on day 1. The patient was intubated on day 9. In view of his poor respiratory status, he was kept under deep sedation using dexmedetomidine, propofol, and midazolam. Rocuronium bromide was also administered. Mobilization was started on day 32. After being weaned from mechanical ventilation, swallowing assessment and training were provided by a speech therapist. He was able to shift from enteral nutrition to an oral diet on day 47. His food intake was normal by the time of discharge. On day 40, his MRC-SS (0-60, none to full) was low at 28. At discharge, he had improved muscle strength in the upper limbs and in the proximal muscles of the lower extremities, his MRC-SS had improved to 34, and his FIM score was 74. ADL, activities of daily living; AFO, ankle-foot orthoses; DEX, dexmedetomidine; FIM, Functional Independence Measure; GS, grip strength; HFNC, high-flow nasal cannula; L, left; MRC-SS, Medical Research Council sum score; MDZ, midazolam; MV, mechanical ventilation; OT, occupational therapy; PT, physical therapy; R, right; ROC, rocuronium bromide; ST, speech therapy; VV-ECMO, veno-venous extracorporeal membrane oxygenation

**Figure 2:**
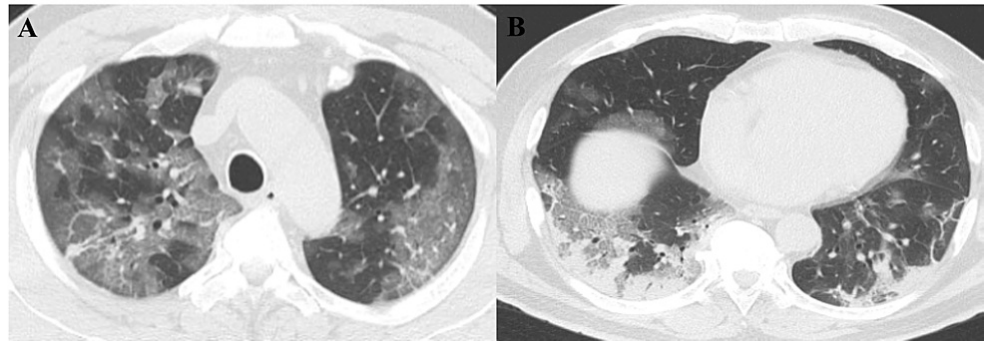
Chest computed tomography on admission Chest computed tomography showed ground-glass shadows and consolidation in both lungs.

His clinical course while in acute care is shown in Figure 1. On admission, he was in respiratory distress with an oxygen saturation of 93% measured in the supine position and was placed on oxygen delivered by high-flow nasal cannula (HFNC) at a concentration of 50% and a flow rate of 30 L/minute. His respiratory rate was 20/minute. Chest images showed ground-glass shadows and consolidation in both lungs (Figure 2). His respiratory status continued to deteriorate. On day 9, endotracheal intubation was performed, and veno-venous extracorporeal membrane oxygenation (VV-ECMO) was started. Thereafter, his respiratory condition improved. On day 25, he was weaned off VV-ECMO, and a tracheostomy was performed. He was discharged from the ICU on day 27. On day 32, he was weaned from mechanical ventilation. Oxygenation was continued until day 41.

Rehabilitative treatment was performed by dedicated staff to minimize the risk of transmission of infection. The patient’s treatment included passive range of motion exercise, respiratory physiotherapy, and prone positioning for six hours/day, even during VV-ECMO. Active mobilization was not possible under mechanical ventilation because the patient’s hypoxemia was too severe and muscle relaxants were needed to prevent lung damage [5]. Muscle-strengthening exercises, active-assisted limb exercises, and training to sit up at the edge of the bed were started when the respiratory status improved.

However, on day 32, despite improvement in the patient’s respiratory status, we noted flaccidity and weakness of the muscles in the extremities on both sides. Voluntary contraction of the tibialis anterior muscle was absent bilaterally. Tendon reflexes in both lower extremities were decreased, and there was a mild decrease in superficial sensation from both dorsal feet to the outer lower legs. These findings led to suspect ICU-AW complicated with bilateral foot drop. Occupational therapy and speech therapy were started on day 41. Upper limb functional training and ADL training were provided by an occupational therapist. Details of the patient’s swallowing assessment and training by a speech therapist are shown in Figure 1. Lower extremity muscle-strengthening training was provided by a physiotherapist. However, the bilateral foot drop did not improve. We then started gait training with ankle-foot orthoses (AFO) to facilitate mobilization. At this stage, we used the standard AFO supplied by the hospital because the prognosis of the foot drop was unknown.

Electrophysiological examinations were performed to investigate the foot drop on day 74. Nerve conduction studies (NCS) showed no compound muscle action potentials in the peroneal nerves on either side and decreased motor conduction velocity and amplitude in the tibial nerve bilaterally (Table 1). Needle electromyography (EMG) revealed neurogenic changes with positive sharp waves and fibrillation, indicating acute denervation in the left tibialis anterior muscle. Polyphasic potential patterns were detected in the lateral head of gastrocnemius, in the short head of the biceps femoris, in the quadriceps femoris, and in the vastus medialis on the left side. The patient could not tolerate NCS in the upper extremities or right lower limb. Accordingly, the diagnosis was polyneuropathy due to ICU-AW complicated with bilateral peroneal nerve palsy. Considering that the immediate recovery of the bilateral peroneal nerve palsy was unlikely, we prescribed custom-made AFO to improve the patient’s ability to undertake gait training.

**Table 1:** Results of nerve conduction studies 3 m, three months after onset; 6 m, six months after onset; ms, millisecond; m/s, meter per second; mV, millivolt; n.e., not evoked

Nerve/site	Months after onset					
	3 m	6 m	3 m	6 m	3 m	6 m
Right tibial	Latency (ms)		Amplitude (mV)		Velocity (m/s)	
Ankle	5.3	6.9	0.49	2.5	37.8	38.3
Popliteal fossa	17.2	17.1	2.06	1.1		
Left tibial						
Ankle	8.7	5.0	2.06	3.01	36.8	34.9
Popliteal fossa	20.4	17.1	2.6	2.58		
Right peroneal						
Ankle	n.e.	n.e.	n.e.	n.e.	n.e.	n.e.
Popliteal fossa	n.e.	n.e.	n.e.	n.e.	n.e.	n.e.
Left peroneal						
Ankle	n.e.	n.e.	n.e.	n.e.	n.e.	n.e.
Popliteal fossa	n.e.	n.e.	n.e.	n.e.	n.e.	n.e.

The patient remained in the hospital for acute care after weaning from mechanical ventilation. During this time, his grip strength, Medical Research Council sum score (MRC-SS) [6], and Functional Independence Measure (FIM) score [7] were regularly assessed. His muscle strength was improved, as shown in Figure 1. However, there was still no voluntary contraction in the tibialis anterior muscle on either side during hospitalization. The motor component of the FIM score at discharge from acute care was 39. He was able to walk about 180 m on the six-minute walk test in the ward while wearing bilateral AFO and using a walker. In these training sessions, his Borg scale score [8] was 11-12 at the start and 15-17 at the end. On day 87, he was transferred to an affiliated convalescent rehabilitation facility (Nishinomiya Kyoritsu Rehabilitation Hospital).

Physiotherapy and occupational therapy were continued during his stay in the convalescent rehabilitation facility and included muscle-strengthening exercises, gait training using AFO and T-cane, and ADL training. About four months after onset, he was discharged from this facility. His ADL performance improved to the modified independence level, and his total FIM score increased to 113 (78 for the motor component and 35 for the cognition component). However, exercise tolerance was low at 346 m for the six-minute walk test and about 17 on the Borg scale immediately after exercise, with strong postexercise fatigue. They were expected to be due to prolonged muscle weakness after infection.

Four months after onset immediately after discharge home, he was referred to the outpatient rehabilitation clinic at our hospital. On the first visit, we set the goal of outpatient rehabilitation to return to his previous work. He worked mostly at a desk and delivered a one-hour lecture once a week while standing. His job included a 30-minute train ride and a 600-m walk to and from the train station, meaning that to return to work, he needed to be able to walk independently for about an hour at an intensity of 3 metabolic equivalents (METs) [9]. We instructed him to perform limb muscle-strengthening exercises (e.g., bicep curl, wall push-off, arm raises, knee straightening, squats, and heel raises; three sets of 10 repetitions of each exercise) and low-intensity walking exercises while monitoring his respiratory and circulatory status. Initial exercise was at 2 METs (walking on level ground at a speed of about 50 m/minute) for 20 minutes/session twice a day, five days a week, with a gradual increase in intensity. We asked him about his training status and adjusted the exercise regimen as necessary.

Table 2 shows the patient’s progress in regaining muscle strength, ADL performance, and six-minute walk distance. It took a long time for the improvement of bilateral foot drop. However, his physical function and ADL gradually improved, and six months after onset, he used the AFO only for walking outside. The motor component of the FIM score improved to 86 and consisted of scores of 6 for toilet transfer, bathtub transfer, walking, climbing stairs, and bowel management and 7 for other motor-related items. Then, he was able to walk for approximately 2 km in 30 minutes using the AFO and T-cane without fatigue, which corresponded to an intensity of 3 METs. The follow-up electrophysiological examination was performed six months after onset, and the results are shown in Table 1. NCS still showed no compound muscle action potentials in the peroneal nerve area. However, the EMG showed a polyphasic pattern in the tibialis anterior muscles, indicating that reinnervation had started. These findings suggested that further functional improvement was possible. No obvious abnormality was found in the upper limbs on EMG.

**Table 2:** Changes in muscle strength, ADL, six-minute walk distance, and exercise load ADL, activities of daily living; AFO, ankle-foot orthoses; FIM, Functional Independence Measure; m/min, meters per minute; MRC-SS, Medical Research Council sum score; R/L, right/Left; 6MWD, six-minute walk distance

Months after onset		1.5 months	3 months	4 months	6 months	18 months
Grip strength (R/L)		7.4 kg/8.8 kg	9.7 kg/15.9 kg	16.7 kg/24.5 kg	24.7 kg/29.8 kg	40.4 kg/43.6 kg
MRC-SS (total score)		28	34	40	52	58
Shoulder abduction (R/L)		3/3	4/4	4/4	5/5	5/5
Elbow flexion (R/L)		3/3	3/3	4/4	5/5	5/5
Wrist extension (R/L)		2/2	2/3	3/3	4/4	5/5
Hip flexion (R/L)		3/3	3/4	4/4	5/5	5/5
Knee extension (R/L)		3/3	4/4	4/4	5/5	5/5
Ankle dorsiflexion (R/L)		0/0	0/0	1/1	2/2	4/4
FIM (motor/cognition)		48 (15/33)	74 (39/35)	113 (78/35)	121 (86/35)	126 (91/35)
6MWD		-	180 m (walking with a walker)	346 m (walking with T-cane and AFO)	402 m (walking with T-cane and AFO)	604 m (walking without T-cane and AFO)
Exercise load at home		-	-	2 METs (walking on level ground at 48-50 m/min）	3 METs (walking on level ground at 64-67 m/min）	4-4.5 METs (walking on plowed field or sand at 65-70 m/min）

About seven months after onset, he was able to return to his full-time work as a civil servant. Thereafter, we continued to instruct low-intensity exercise and muscle-strengthening training, as well as assessment of the need for continued use of walking aids. Finally, 18 months after onset, he was able to walk outside without AFO and T-cane, and his muscle strength including ankle dorsiflexor significantly improved. The six-minute walk distance was 604 m, and the postexercise Borg scale was 11. He was aware that he had improved to the same level of physical function before onset.

## Discussion

This report describes a patient with severe COVID-19 who required mechanical ventilation and ECMO and subsequently developed ICU-AW with bilateral peroneal nerve palsy. We predicted the functional outcome from the results of an electrophysiological examination and prescribed AFO for gait training from the acute stage. We also set rehabilitation goals after a detailed assessment of the requirements of his occupation and provided a step-by-step training exercise regimen for his return to work. Then, we continued to follow up to return to life equivalent to that before the onset of COVID-19. Our regimen was successful, and he returned to his previous work and life as before onset.

ICU-AW has been reported to occur in 27%-72% of patients with COVID-19 [10]. Muscle weakness caused by ICU-AW after COVID-19 persists even at six months after the onset of COVID-19, especially in severe cases [11]. Moreover, our patient developed bilateral peroneal nerve palsy secondary to COVID-19. The common cause of peroneal nerve palsy associated with COVID-19 is reported due to compression from prone positioning or prolonged supine position, and other causes include direct viral infiltration of the peripheral nerve and immunologic mechanisms [12,13]. Electrophysiological examinations such as NCS and EMG allow accurate assessment of the severity of these neuromuscular involvements in patients with COVID-19 and can be used to predict the functional outcome [14,15]. In this situation, specific rehabilitative treatments should be implemented to allow the best chance of recovery [14]. Shabat et al. reported that the amplitude in the tibial and peroneal nerves on NCS was correlated with the duration of rehabilitation and the motor component of the FIM at discharge [15]. Moreover, Wallerian degeneration and the onset of denervation potentials on EMG are clinically correlated, and this information is used to prognosticate the time course and extent of recovery in response to therapeutic interventions [16]. In our case, NCS showed reduced amplitude in both tibial nerves, and no conduction was induced in both peroneal nerves. The EMG findings suggested denervation, indicating that immediate recovery was unlikely. Therefore, we prescribed customized AFO starting during the acute hospitalization period.

Return to work is one of the goals of rehabilitation. In a previous study, patients with COVID-19 who spent more than 14 days on a ventilator had only a 67% rate of return to work six months after discharge. Even patients who can return to work have reported reduced work performance [17]. Therefore, it is necessary to understand in detail each patient’s work style, workload, and working environment and to monitor after return to work. However, few studies have investigated a rehabilitative approach for patients recovering from critical illness after discharge home. In this case, fatigue after exercise due to muscle weakness was a barrier to returning to work. We set exercise load and ADL targets based on detailed information about the patient’s work and commuting requirements. Given that frequent hospital visits may be a potential barrier in terms of locomotive load for participants in training programs [18,19], less frequent hospital visits with ongoing periodic evaluation and well-managed home exercise could be an alternative [20]. Accordingly, after discharge home, we provided outpatient rehabilitation with detailed instructions for home-based exercise so that the patient could manage himself with the goal of returning to his usual work and life. Based on the results of clinical and electrophysiologic evaluation six months after onset, follow-up was continued after return to work. This individually tailored and continuous rehabilitative regimen was successful in our case.

This report has some limitations. First, the patient could not tolerate a full electrophysiological examination during his acute hospitalization. Therefore, we clinically suspected that the cause of the foot drop was due to nerve entrapment due to prone positioning as reported in the past [12,13], but we were unable to confirm this. Second, the exercise intensity during the outpatient rehabilitation period was determined by the patient’s subjective reports and not by specific cardiopulmonary examinations (e.g., cardiopulmonary exercise testing). Third, it was not possible to assess his physical function before the onset of COVID-19. However, despite these limitations, a detailed assessment of our patient’s work requirements and a well-managed rehabilitative regimen allowed him to return to work and recover his ADL performance and physical function.

## Conclusions

COVID-19 has been reported to be associated with various neuromuscular manifestations. Electrophysiological studies are important for the prediction of functional prognosis and goal setting for rehabilitative treatment. In addition, a comprehensive rehabilitation approach from the acute phase to post-discharge home is necessary to deal with the long-term sequelae of severe COVID-19.
